# Supplementary material for: The Prognostic Value of Anemia in Patients with Preserved, Mildly Reduced and Recovered Ejection Fraction
Source: Diagnostics (Basel). 2022 Feb 17;12(2):517. doi: 10.3390/diagnostics12020517 (PMC8871183; doi:10.3390/diagnostics12020517)
Supplement: Supplementary file 1 [file diagnostics-12-00517-s001.zip › diagnostics-1583713-supplementary.pdf]

diagnostics

MDPI

Supplementary Figures

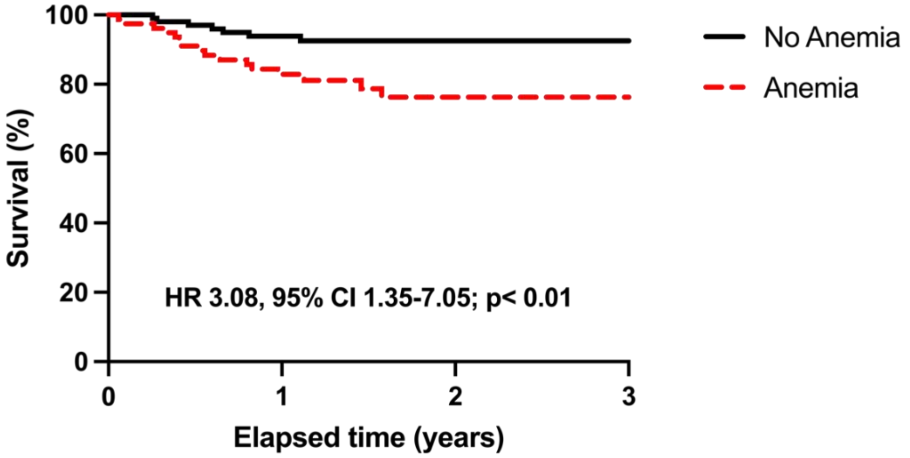

Patients at risk				
Anemia	78	52	13	10
No Anemia	101	78	15	11

a

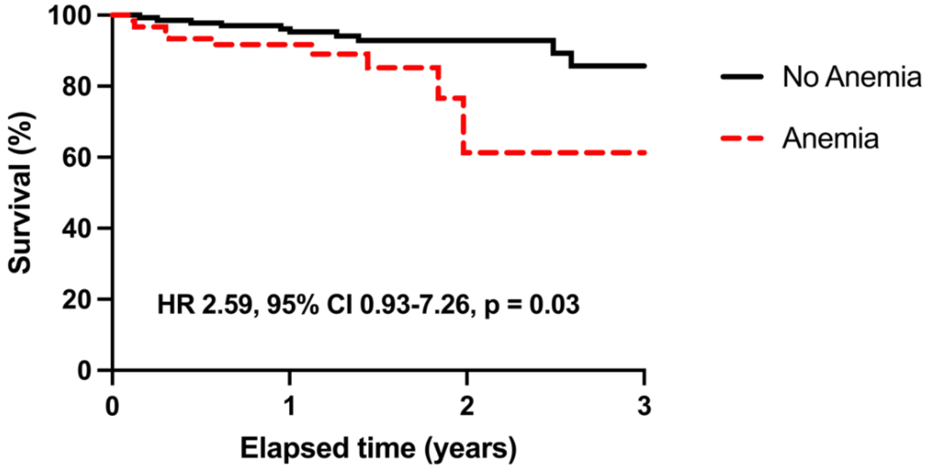

Patients at risk				
Anemia	60	45	4	3
No Anemia	136	107	29	24

b

Figure S1. a. Kaplan-Meier Estimates of the Probability of Survival according to presence vs. absence of anemia in males; **b.** Kaplan-Meier Estimates of the Probability of Survival according to presence vs. absence of anemia in females.

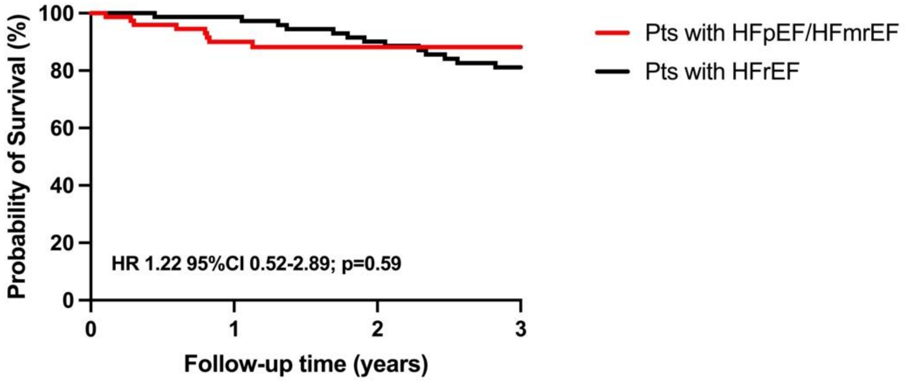

Patients at risk				
Pts with HFpEF/HFmrEF	75	54	9	8
Pts with HFrEF	75	74	61	48

Figure S2. Kaplan-Meier Estimates of the Probability of Survival in the total HFpEF-HFmrEF and HFrecEF groups.
